# Redox Balance in β-Thalassemia and Sickle Cell Disease: A Love and Hate Relationship

**DOI:** 10.3390/antiox11050967

**Published:** 2022-05-13

**Authors:** Rayan Bou-Fakhredin, Lucia De Franceschi, Irene Motta, Assaad A. Eid, Ali T. Taher, Maria Domenica Cappellini

**Affiliations:** 1 Department of Clinical Sciences and Community Health, University of Milan, 20122 Milan, Italy; rayan.boufakhredin@unimi.it (R.B.-F.); irene.motta@unimi.it (I.M.); 2 Department of Medicine, University of Verona, and Azienda Ospedaliera Universitaria Verona, 37128 Verona, Italy; lucia.defranceschi@univr.it; 3 UOC General Medicine, Fondazione IRCCS Ca’ Granda Ospedale Maggiore Policlinico, 20122 Milan, Italy; 4 Department of Anatomy, Cell Biology and Physiological Sciences, Faculty of Medicine, American University of Beirut, Beirut 1107 2020, Lebanon; ae49@aub.edu.lb; 5 Division of Hematology-Oncology, Department of Internal Medicine, American University of Beirut Medical Center, Beirut 1107 2020, Lebanon; ataher@aub.edu.lb

**Keywords:** β-thalassemia, sickle cell disease, oxidative stress, oxidant, antioxidant, redox, reactive oxygen species

## Abstract

β-thalassemia and sickle cell disease (SCD) are inherited hemoglobinopathies that result in both quantitative and qualitative variations in the β-globin chain. These in turn lead to instability in the generated hemoglobin (Hb) or to a globin chain imbalance that affects the oxidative environment both intracellularly and extracellularly. While oxidative stress is not among the primary etiologies of β-thalassemia and SCD, it plays a significant role in the pathogenesis of these diseases. Different mechanisms exist behind the development of oxidative stress; the result of which is cytotoxicity, causing the oxidation of cellular components that can eventually lead to cell death and organ damage. In this review, we summarize the mechanisms of oxidative stress development in β-thalassemia and SCD and describe the current and potential antioxidant therapeutic strategies. Finally, we discuss the role of targeted therapy in achieving an optimal redox balance.

## 1. Introduction

The oxidative status of cells is dependent on the balance between oxidants and antioxidants. This balance is crucial to achieving normal physiology and maintaining cellular homeostasis. Thus, the thinning control of reactive oxygen species (ROS) is extremely important since low levels of ROS might participate in the signaling pathways involved in the differentiation and proliferation of the erythroid precursors [1,2,3,4].

Oxidative stress can manifest in several pathologies when the balance between oxidants and antioxidants is broken, as evident in β-thalassemia and sickle cell disease (SCD). Excessive levels of ROS can lead to cytotoxicity as these radicals often bind to cellular components such as proteins, membrane lipids, and DNA. Different mechanisms exist behind oxidative stress development. These include the accumulation of α-globin chains and free iron as seen in β-thalassemia or the cyclic polymerization/depolymerization of hemoglobin S (HbS) as seen in SCD. Thus, the activation and expression of potent anti-oxidant machinery are required to ensure the proper maturation of erythroid precursors and the survival of red blood cells (RBCs) in the peripheral circulation [5,6]. While more studies are necessary to better understand the role and mechanism of action of antioxidant agents, they have shown to be effective in improving the pathological manifestations of β-thalassemia or SCD by re-balancing the cellular redox state. Different molecules have been tested and these have acted as endogenous or as exogenous antioxidants that scavenge and inactivate ROS, leading to cellular protection against oxidation.

Progress in the knowledge of oxidation and cellular damages in β-thalassemia and SCD may lead to the identification of new antioxidant therapies that can prevent or delay the development of organ complications behind erythroid cells in patients. In this review, we summarize the mechanisms of oxidation in β-thalassemia and SCD and describe the current and potential antioxidant therapeutic strategies. Finally, we discuss the role of targeted therapy in achieving an optimal redox balance in these chronic invalidating disorders.

## 2. Evolutionary Perspective of a Redox Balance

Reactive oxygen species are chemically reactive molecules that are formed as a by-product of different cellular metabolic reactions. They include hydrogen peroxide (H_2_O_2_), superoxide free radicals (O_2_^•−^), as well as nitrogen-based free radical species such as peroxynitrite (ONOO^−^) or nitric oxide (NO) [7,8]. Reactive oxygen species are significant cellular entities that play a role in cellular proliferation, signal transduction, host defense mechanisms, homeostatic preservation, and gene expression [9].

Being heritable biological adaptations, ROS have evolved concurrently with natural and environmental modifications. Such modifications have been crucial for better understanding their role and mechanisms of action while providing insights into the evolutionarily preferred mechanistic physiologies of cells. One strategy of major significance in physiological functions is that cellular events revolve around fixed and coordinated set points. This phenomenon is physiologically referred to as homeostasis, and ROS are tightly counterbalanced by various cellular antioxidants to promote optimal redox homeostasis [10]. In fact, all cells possess effective antioxidant mechanisms that neutralize and remove ROS. Moreover, several enzyme systems present in our bodies neutralize ROS by metabolic conversion [11].

Reactive oxygen species are recognized as specific mediators and second messengers of cell signaling that play a role in vascular tone, immune responses, cell protection, and hormonal actions [12,13,14]. In sync with this homeostatic balance, these functions are primarily maintained and counter-balanced by anti-oxidative mechanisms that regulate the bioavailability of oxidative species. However, under pathological conditions, oxidant radicals lead to a state of oxidative stress. In cells, this results in the malfunctioning of many organelles, particularly the membrane, and may lead to cytotoxicity and eventually organ damage and failure [8].

Several reports have indicated increased ROS levels to be directly correlated with the irreversible oxidation of cellular components that eventually contribute to cellular dysfunction and necrosis [15]. Many studies have validated the implications of redox alternations in distinct pathologies such as diabetes and cancer. However, attempts to reduce bioactive ROS to very minimal levels have been shown to be detrimental because in lower concentrations these ROS can act as signaling molecules [16]. This highlights the significance of the redox system in cellular physiology and homeostatic balance between oxidant and antioxidant molecules [17]. Extensive research is currently aiming at identifying specific cellular sources of ROS production and those that are specifically altered in a cell- and disease-specific manner.

## 3. Sources of Reactive Oxygen Species in Red Blood Cells

Erythroid precursors and erythrocytes are unique cells since they are exposed to cyclic oxygenation/deoxygenation (auto-oxidation) events as long-term survival cells, and they require iron for hemoglobin (Hb) synthesis. Thus, anti-oxidant and cytoprotective systems are crucial for erythroid cell homeostasis. The process of auto-oxidation (from oxygenated Hb to methemoglobin) plays a major role in the generation of ROS inside RBCs [18,19]. This process is characterized by the rapid conversion of O_2_^•−^ to H_2_O_2_ [19,20]. In β-thalassemia and SCD RBCs, Hb auto-oxidation is more pronounced as the Hb molecules in these diseases are highly unstable [19]. Moreover, in SCD, the Hb auto-oxidation process is further exacerbated under hypoxic conditions in the microcirculation and leads to the formation of unstable dimers at reduced Hb concentrations [19].

**Figure 1:**
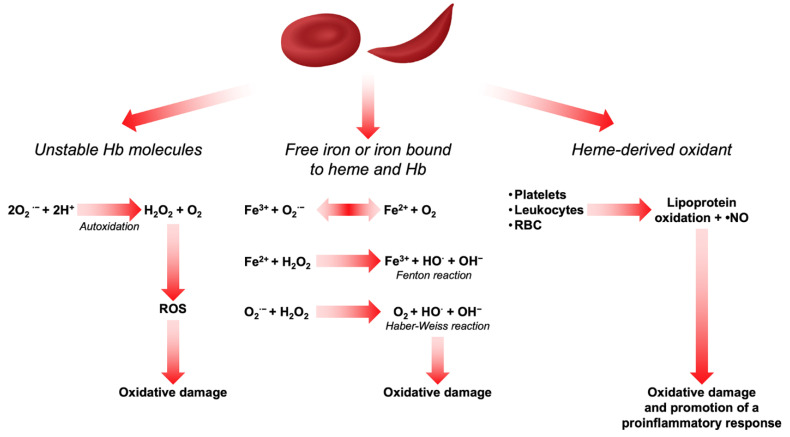
Sources of ROS in RBCs.

Heme and iron are also highly oxidizing agents and sources of ROS in RBCs. Whether iron is in its free form or bound to heme and Hb, it can act as a Fenton reagent in the Haber–Weiss cycle, thereby generating hydroxyl radicals (^•^OH) and promoting extensive oxidative damage [21,22]. Being a hydrophobic molecule, heme interacts with proteins and membrane lipids and thus promotes a series of oxidation reactions [23]. On the other hand, free iron bound to the RBC membrane has been described to participate in the oxidation of membrane proteins and lipid components, thereby affecting their mechanical properties. This increases erythrophagocytosis and contributes to the reduction in RBC survival in peripheral circulation [24,25,26,27]. In β-thalassemia and in SCD, chronic hemolysis overcomes physiologic buffer systems such as haptoglobin and hemopexin and results in increased plasma free Hb and heme [28,29]. Oxidants derived from heme can induce the recruitment of platelets, leukocytes, and RBCs to the vessel wall and produce lipoprotein oxidation and consume ^•^NO to form strong oxidants [30]. In vascular endothelial cells, this activates the transcription factor nuclear factor-κB (NF-κB) which is redox-sensitive and promotes a proinflammatory response by binding to receptors, enzymes, and transcription factors that alter cell metabolism, cell function, and gene expression [31]. All the above-mentioned mechanisms of ROS production are depicted in Figure 1.

**Figure 2:**
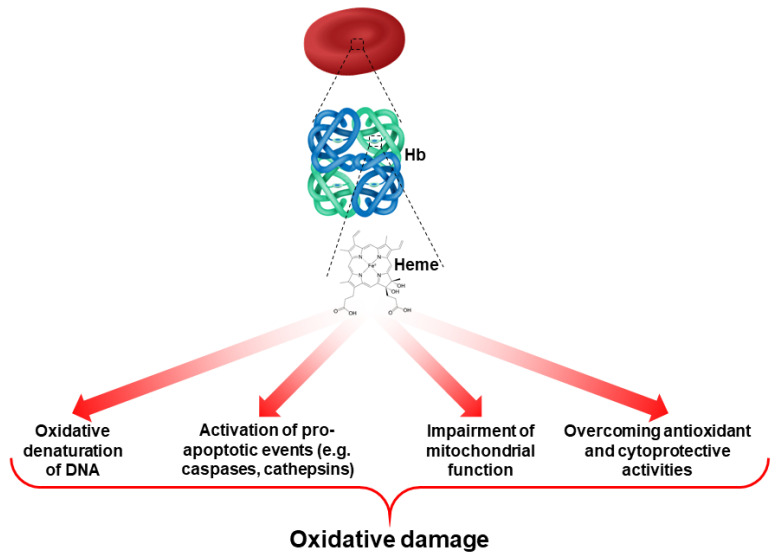
Role of heme as a contributor to ROS production and oxidative damage in RBCs.

In normal erythropoiesis, heme biosynthesis is a key event in Hb production. In β-thalassemic erythropoiesis, different studies have shown an early phase of heme accumulation, followed by the activation of antioxidant and cytoprotective systems that generate a second phase of relative heme deficiency [3]. This is important since heme can act as a source of ROS in RBCs and cause oxidative damage [19,32,33] (Figure 2). Thus, prolonged and severe oxidation, due to the combination of abnormal heme biosynthesis and the accumulation of alpha-globin chains play a crucial role in the ineffective erythropoiesis of β-thalassemia.

## 4. Oxidative Damage to Intracellular Components in Red Blood Cells

### 4.1. Oxidative Damage to Membrane-Cytoskeleton Proteins

Oxidative stress also has an effect on the overall cytoskeletal network and its associated proteins [34,35]. One study conducted on β-thalassemia RBCs showed that spectrins might be targeted by oxidation. This perturbates their interactions with other cytoskeletal proteins such as actin or with multiprotein complexes bridging the membrane to the cytoskeleton such as protein 4.1 or band 3 [35]. Consequently, impaired stability in the interaction between the cytoskeleton and the cellular membrane becomes evident. It is noteworthy to mention that the oxidation-induced and abnormal clusterization of band 3 contributes to increased RBC fragility and the generation of erythroid microvesicles which contributes to the pro-coagulant phenotype of both β-thalassemia and SCD [24,36,37].

Several cytoskeletal proteins in RBCs derived from SCD patients have been shown to undergo oxidation-mediated post-translational protein modifications (PTPM). Additionally, RBCs from transgenic SCD mice revealed that these irreversible PTPM detected in HbS molecules were found in the β-chain and included the ubiquitination of Lys96 and Lys145 and the irreversible oxidation of Cys93 and [38,39]. This ubiquitination process in HbS molecules occurs as a result of the accumulation of oxidatively damaged HbS molecules in RBCs as well as in microparticles and could likely be due to the redox imbalance-dependent proteasomal inhibition in SCD. A recent study conducted on RBCs and microparticle proteomes from SCD patients showed increased ubiquitination and phosphorylation of cytoskeletal proteins when compared to control cells [39]. Remarkably, these PTPM have been identified in spectrin, ankyrin, band 3, carbonic anhydrase, and band 4.1 [39]. Moreover, an increase in ROS production by auto-oxidized HbS led to an increase in the accumulation of oxidative lesions by membrane components. These, in turn, can degrade polyunsaturated lipids and thus lead to the formation of malondialdehyde as a by-product and damage proteins localized in the region near membrane-associated Hb [40]. Among PTPM, oxidation might also activate tyrosine kinases such as Syk or Lyn, a kinase of the Src family. Both Syk and Lyn target RBC membrane proteins such as band 3, contributing to both protein conformation state and protein–protein interaction [2,25,36,41,42]. This is important for the mechanical properties of the membrane and the volume/surface regulation of pathologic RBCs.

### 4.2. Oxidative Damage to Membrane Lipids

Lipid peroxidation and protein oxidation disturb the organization of the lipid mem-brane and lead to cellular deformability [43]. This disruption causes phosphatidylserine (PS) to be exposed to the outer membrane of the cell which in turn signals macrophages to engulf and degrade the PS-exposed RBCs [44], a phenomenon seen in β-thalassemia patients [44]. The exposure of PS and subsequent macrophage degradation is the mechanism of RBC removal during eryptosis. Eryptosis is the suicidal death process of RBCs that occurs prior to senescence and after injury. This phenomenon is characterized by cell shrinkage and the loss of membrane organization and can be further exacerbated by several factors, including oxidative stress.

As for SCD RBCs, the high rate of intracellular ROS production in addition to the presence of auto-oxidizing HbS in the cell membrane leads to the oxidative damage of membrane lipids and the loss of membrane lipid asymmetry. This alters membrane surface properties and permeability and exposes PS [45]. The externalization of PS is a critical event in the disease progression [46]. It is considered a key step in the premature senescence process, favoring the removal of RBCs from circulation and the release of erythroid microvesicles. This leads to a state of chronic anemia as well as endothelial dysfunction in SCD [47]. The pathophysiology of SCD is also characterized by increased plasma levels of secretory phospholipase A2 (sPLA2), a powerful inflammatory mediator that can selectively hydrolyze phospholipids in RBCs exposing PS, promoting their hemolysis [48,49]. The activation of sPLA2 also generates phospholipid breakdown products that can affect overall endothelial function. Moreover, PS-exposing RBCs can also activate phospholipase D, which catalyzes the hydrolysis of phosphatidylcholine into phosphatidic acid and choline [50]. 

## 5. A Focus on Oxidative Stress in β-Thalassemia

### 5.1. Oxidative Stress and Hemoglobin

β-thalassemia is characterized by the unbalanced production of globin chains, resulting in excess free α-globins [51]. These unstable α-globins tend to auto-oxidize, denature, and precipitate as hemichromes [52,53]. Hemichromes then bind to the cytoplasmic domain of band 3 and mediate the oxidative cross-linking through disulfide bonds. Subsequently, both heme and free iron are released and globin proteins precipitate. This initiates a self-amplifying redox reaction that oxidizes additional Hb molecules, depletes cellular reduction potential, and triggers phosphorylative responses, which lead to membrane destabilization and the acceleration of RBC removal by splenic macrophages [51,54,55]. In fact, deoxyhemoglobin (deoxyHb) binds avidly and reversibly to band-3 [56]. Because band 3 is associated with multiple metabolic, solute transport, signal transduction, and structural proteins, the oxygen-dependent regulation of erythrocyte properties is mediated by the reversible association of deoxyHb with band 3 [56]. 

### 5.2. Oxidative Stress and Ineffective Erythropoiesis

Ineffective erythropoiesis is one of the main pathophysiological culprits in β-thalassemia. The impaired ratio between α and β subunits results in the accumulation of unbounded α-chains during erythroblast maturation. These bind heme and eventually form hemichromes, which precipitate and bind to the plasma membrane [57,58]. Evidence of oxidative stress due to ineffective erythropoiesis comes from studies conducted on bone marrow cells and in developing thalassemia erythroid precursor cells. Many studies conducted with bone marrow cells of β-thalassemia patients have indicated an increased number of activated macrophages, which might cope with the several damaged erythroblasts [59,60]. Interestingly, one study showed that the presence of α-globin precipitates in cells at the polychromatophilic erythroblast stage of maturation, a stage characterized by increased hemoglobinization and apoptosis in β-thalassemia erythropoiesis [61,62]. Another study conducted in a mouse model of β-thalassemia intermedia (β-TI) showed that growth differentiation factor 11 (GDF11), a transforming growth factor β (TGF-β) superfamily ligand, blocks the terminal differentiation of erythroid precursors by promoting oxidative stress and α-globin precipitation [63]. The expression of GDF11 is also induced by oxidative stress, which indicates the presence of an autocrine amplification loop that can promote ineffective erythropoiesis [63]. Oxidative stress in developing thalassemia erythroid precursors has also been associated with increased apoptosis, as manifested by the externalization of PS. This notion suggests that oxidative stress can also be responsible for the ineffective erythropoiesis itself [64].

### 5.3. Oxidative Stress and Iron Overload

In β-thalassemia, iron overload is one of the most common disease-related complications and is a major cause of morbidity and mortality [65,66]. In the plasma, iron is normally bound to transferrin. In β-thalassemia patients, however, iron overload saturates the ability of the transferrin iron transport system, leading to the formation of non-transferrin bound iron (NTBI) and labile plasma iron (LPI). Both NTBI and LPI circulate in plasma and subsequently become deposited inside the susceptible cells [67,68]. The long-term uptake and accumulation of these molecules can lead to high levels of storage iron and labile cellular iron [69]. When the scale of the cellular production of the labile iron pool exceeds the capacity of the cell to synthesize new ferritin molecules, a critical concentration is reached. This intracellular labile iron pool is redox-active, catalyzing the Fenton and Haber-Weiss reactions, thus generating ROS [70]. The production of ROS due to iron overload by the metabolism of NTBI plays an essential role in inducing cellular dysfunction, apoptosis, and ferroptosis [71,72,73,74]. For example, one study showed that NTBI-triggered iron overload can aggravate atherosclerosis in ApoE-/- FPNwt/C326S mice, suggesting a pro-atherogenic role for iron [75]. This finding was characterized by endothelial permeabilization, activation, dysfunction, elevation in pro-inflammatory mediators, and the induction of highly vulnerable plaques [75]. 

## 6. A Focus on Oxidative Stress in Sickle Cell Disease

### 6.1. Connection between Oxidation and Oxidases in SCD

In the vascular compartment of patients with SCD, the overactivation of nicotinamide adenine dinucleotide phosphate (NADPH) oxidase, xanthine oxidase (XO), and uncoupled nitric oxide synthase (eNOS) can generate ROS [76,77,78,79]. NADPH oxidase is the major O_2_^•−^-producing enzyme in RBCs, vascular endothelial cells, and leucocytes. The production of ROS by NADPH oxidase can lead to a state of hemolysis that is often associated with infections or vessel-occlusive crises [78]. The O_2_^•−^ derived from this process contributes to the pro-thrombogenic and pro-inflammatory responses that are often associated with SCD [24]. Intracellularly, NADPH oxidase activity is regulated by protein kinase C and Rac GTPases in RBCs. Extracellularly, however, it is regulated by signaling factors such as TGF-β1 and endothelin-1 [79]. NADPH oxidase-induced ROS may cause direct oxidative damage to several subcellular structures. This reduces the deformability of RBCs and results in increased fragility and hemolysis [79]. On the other hand, XO is considered to be another major source of O_2_^•−^ and H_2_O_2_ in RBCs. In SCD patients, the activity of XO is often increased in the plasma. Episodes of hypoxia/reoxygenation in SCD patients can excite the release of this enzyme from the liver and into the circulation. These circulating XO molecules can then bind to vessel luminal cells, impair vascular function, and create an oxidative setting [25].

### 6.2. Oxidative Stress and Reduced ^•^NO Bioavailability in SCD

SCD is characterized by a local reduction in NO, resulting in vascular dysfunction and abnormal vascular tone [80,81]. This occurs as a result of intravascular hemolysis and the consumption of physiologic buffer systems such as haptoglobin and hemopexin. Indeed, free heme and free Hb bind to NO, promoting the local reduction in NO availability [82,83,84,85]. The reaction of ^•^NO with oxygenated Hb results in the formation of methemoglobin and nitrate [86]. Additionally, superoxides bind to NO to produce ONOO^−^. This contributes to a decrease in NO levels and leads to further ROS production [30,87]. Another NO-derived metabolite known as nitrite is consumed by the heme-containing myelop-eroxidase. Often found in neutrophils, this myeloperoxidase enzyme catalytically reacts with nitrite in the presence of H_2_O_2_ and generates powerful radicals such as nitrogen di-oxide (^•^NO_2_), which can create a state of oxidation [88].

In SCD, the release of arginase during hemolysis can also contribute to low NO levels. Arginase competes with nitric oxide synthase (NOS) for its substrate, L-arginine, which it uses to produce NO and L-citrulline [77]. The absence of tetrahydrobiopterin (BH4), due to peroxynitrite production, or L-arginine leads to the “uncoupling” of NOS and results in further ROS production in SCD [77,89]. This condition diminishes ^•^NO bioavailability in both SCD and other vasculopathies [90].

Asymmetric dimethylarginine (ADMA) is the major endogenous inhibitor of NOS that competes with L-arginine for NOS [91,92]. ADMA can lead to the uncoupling of all NOS isoforms by converting them from ^•^NO-producing enzymes to enzymes generating O_2_^•−^ and other derived oxidant products, thus leading to an increase in oxidative stress and decreasing ^•^NO bioavailability [93,94]. When plasma concentrations of ADMA are elevated, this can be a risk factor for the development of endothelial dysfunction and is used as a predictor for all-cause and cardiovascular mortality [95]. In SCD patients, increased plasma levels of ADMA have been reported. These were not only correlated with hemolytic markers but also associated with increased amounts of soluble vascular cell adhesion molecule-1 (sVCAM-1), SCD-related pulmonary hypertension, and early death [96,97,98].

## 7. Antioxidant Enzymes and Cytoprotective Defenses in β-Thalassemia and SCD

In the previous sections, we discussed how oxidation contributes to red cell damage and ineffective erythropoiesis in both β-thalassemia and SCD. Here, we focus on endogenous antioxidant and cytoprotective systems, which are crucial to limit prolonged oxidation and ensure cell survival.

### 7.1. Cytoprotective and Anti-Oxidant Systems in Erythroid Cells

#### 7.1.1. Peroxiredoxin-2

Peroxiredoxins play a significant role in RBCs through their antioxidant properties and chaperone function. Proteasomes are multi-catalytic complexes with important roles in protein control. Their activity in stored RBCs is affected by both storage time and the donor’s characteristics. In fact, some recent studies on patients with β-thalassemia trait and SCD have provided significant information about the interaction of peroxiredoxins and the RBC proteasomal machinery [99,100,101,102]. Of interest is peroxiredoxin-2 (Prx2), one of the most abundant proteins in RBCs, which plays a major role against oxidation [24,27,103,104,105,106,107,108,109]. It is a member of the typical homodimeric 2-Cys Prxs, the wider group that uses two cysteine residues to detoxify many organic peroxides, including H_2_O_2_ and ONOO^−^ [2,103,110]. The tyrosine phosphorylation of Prx2 increases its activity in response to severe oxidation [2]. In addition to its enzymatic function, and under oxidative stress conditions, peroxiredoxins have been shown to acquire a chaperone function, allowing them to migrate to the membrane and interact with numerous proteins [111,112]. The importance of Prx2 in RBCs and erythropoiesis has been supported by results in mouse models genetically lacking Prx2 (Prx2-/-), which display age-dependent chronic hemolysis and ineffective erythropoiesis [107,113]. We have recently shown that Prx2 expression is increased in β-thalassemic RBCs and plays an important role during both normal and pathologic erythropoiesis to support erythroid maturation against oxidative stress [25,114]. In addition, Prx2 acts as a backup mechanism in the presence of severe oxidation due to iron overload. This highlights the importance of Prx2 in chronic hemolytic anemia such as β-thalassemia [104,107]. In fact, one recent study showed that peroxiredoxin 1 cooperates with Prx2 in the antioxidant pathways of erythroid cells in patients with β-TI [115].

In SCD, Prx2 has been also shown to be involved in the dynamic cross-talk between the cytoplasm compartment and the RBC membrane of SCD mice exposed to hypoxia-reoxygenation stress, a condition used to mimic acute sickle cell-related vaso-occlusive crisis (VOC) [101,102,116]. Indeed, Prx2 membrane association might decrease local oxidation, preventing band 3 clusterization and the generation of erythroid microvesicles.

#### 7.1.2. Superoxide Dismutase and Catalase

In RBCs, superoxide dismutase (SOD) acts as a first-line defense mechanism against free radicals. This cytosolic enzyme that contains both copper and zinc converts O_2_^●−^ into the less reactive H_2_O_2_ [117]. Found at high concentrations in RBCs, catalase is an intracellular enzyme that protects cells and tissues from the toxic effects of H_2_O_2_ [118]. A major increase in SOD was found in beta-thalassemia major (β-TM) patients when compared to healthy controls [119]. Another study, however, showed no significant change in the levels of SOD and catalase in patients with β-thalassemia [120]. As for SCD, one study by Antwi-Boasiako et al. reported that levels of SOD and catalase in RBCs were significantly lower in SCD patients when compared to healthy controls [121]. This is in agreement with other studies that also showed low levels of antioxidant enzymes in SCD patients [122,123,124]. A previous study conducted by Manfredini et al., however, reported significantly higher levels of SOD in the RBCs of SCA patients compared to their healthy counterparts [125]. In the same study, levels of catalase were not found to be significantly different between the SCA and controls. Moreover, HbSS patients with VOCs had significantly lower levels of SOD in RBCs when compared to the other study subjects. The excessive amount of ROS produced during a VOC may have partly contributed to the lower levels of SOD and catalase in these patients. Findings from a 2019 study by Antwi-Boasiako et al. also proposed that the low levels of SOD and catalase in RBCs seen in SCD patients with the SS genotype may be due to the significant depletion of antioxidants, such as nitric oxide and vitamins [121]. As evident by many of these studies, there has been a discrepancy in reports on SOD and catalase activity. Increased levels of antioxidant enzymes such as SOD and catalase may be seen in various settings including an acute inflammatory phase, a state of trauma, and upon exposure to increased levels of pro-oxidants [126]. Decreased SOD levels could be due to increased oxidative stress which results in excessive antioxidant consumption and thus antioxidant deficiency [127]. On the other hand, a decrease in catalase activity might be due to the chronic level of oxidative stress itself, whereas an increase in its activity might be due to a protective measure by the body to scavenge ROS. Increased catalase levels could also be a consequence of higher reticulocyte content in SCD patients, for example [128]. Consequently, further studies with a much larger sample size and unified methodological approach are needed to better understand the reasons behind the varying levels of SOD and catalase that have been reported in β-thalassemia and SCD patients.

#### 7.1.3. The Glutathione System

The glutathione system (GSH) is an important scavenger of free radicals and a potent endogenous antioxidant that can protect cells from oxidative injury [129,130]. In β-TM patients, higher levels of glutathione peroxidase (GPx) were observed as compared to healthy controls [119]. Another study, however, reported the levels of the antioxidant enzyme GPx to be significantly lower in β-thalassemia patients [131]. These findings are in agreement with the study of Garelnabi et al., which showed that the low levels of GPx in children with β-thalassemia seem to result from the enzyme inhibition or reduced activity due to the excessive production of H_2_O_2_ [132]. In a study on SCD patients, the oxidative status of RBCs was evaluated by exploring the glutathione system. The overall total content of GSH and reduced GSH in SCD RBCs was 32–36% lower in these patients [133]. This finding is in line with other previous studies and further highlights this increased oxidative stress status that is characteristic of sickle RBCs [134,135,136]. The same study also reported higher GPx activities in SCD patients compared to controls, and no significant differences in the activity of glutathione reductase activity among the studied cohorts [133]. The contradictory conclusions of these studies might be related to patient selection (age, presence/absence of the spleen, kidney disease), reticulocyte count, and/or the number of circulating erythroblasts. Moreover, the enzymatic levels/activity of the glutathione system is higher in young RBCs compared to mature/old RBCs.

### 7.2. Cytoprotective Systems in Erythropoiesis

#### 7.2.1. Heme-Regulated Inhibitor of Protein Translation

In β-thalassemic erythropoiesis, the thinning control of iron and heme is a priority for cell survival. Heme-regulated inhibitor of protein translation (HRI) has been shown to repress globin translation in heme-deficient erythroid precursors [137]. HRI is the heme-regulated eukaryotic initiation factor-2α (eIF2α) kinase that phosphorylates a subunit of eIF2. In vitro studies have shown that HRI activation also involves ROS and necessitates the presence of the heat shock proteins 70 and 90 [138]. In β-thalassemic mice that genetically lack HRI, a more severe hematological phenotype was shown when compared to normal β-thalassemia mice, supporting the key role of eIF2α in stress erythropoiesis [139]. Translational up-regulation of activating transcription factor 4 (ATF4) mRNA by the HRI-eIF2αP signaling pathway was necessary to mitigate oxidative stress and promote erythroid differentiation [140]. The repression of globin mRNA translation by HRI decreased proteotoxicity and allowed the ATF4 protein to be expressed. This is an important process in terminal erythropoiesis to maintain pivotal mitochondrial functions and oxidative homeostasis [141].

#### 7.2.2. Heme Oxygenase-1 (HO-1)

The catabolism of heme is also important in normal and pathologic erythropoiesis. HO-1 (heme oxygenase-1) is an enzyme that catalyzes the degradation of heme [142]. It is generally considered to be a protective enzyme because of its ability to breakdown the pro-oxidant “free” heme and release biliverdin and bilirubin, which exhibit antioxidant properties. Thus, HO-1 might represent a potential therapeutic target in ineffective erythropoiesis and thus improve oxidative stress in β-thalassemia. In fact, the administration of tin-protoporphyrin IX, an HO-1 inhibitor, improved overall hematological parameters and decreased anemia, ineffective erythropoiesis, spleen size, liver iron, and erythropoietin levels, and increased the hepcidin serum levels in Th3/+ mice. Treatment with tin protoporphyrin IX also decreased apoptosis, increased RBC lifespan, and reduced ROS levels [143]. 

#### 7.2.3. Alpha Hemoglobin-Stabilizing Protein

Among atypical chaperone systems, the α hemoglobin stabilizing protein (AHSP) has been reported to play an important role in β-thalassemic erythropoiesis. AHSP binds to α-Hb, prevents its precipitation, and limits free α-Hb toxicities. Alpha Hb bound to AHSP is more resistant to oxidant-induced precipitation and the phenotype of β-TI mice has been shown to be exacerbated by the concomitant loss of AHSP [144]. Moreover, AHSP knock-out mice showed pathological features and a degree of ineffective erythropoiesis that is similar to that seen in β-thalassemia [145]. One cross-sectional study on 37 patients with β-thalassemia and 12 sickle cell anemia (SCA) patients showed that AHSP levels were significantly higher in patients with SCA compared to those with β-thalassemia [146]. Moreover, no significant differences in the level of AHSP were seen between patients with β-TM and β-TI [146].

## 8. Antioxidant Therapeutic Agents

Because oxidative stress plays a significant role in the pathophysiology of diseases such as β-thalassemia and SCD, numerous molecules and pharmacological agents with antioxidant properties have been used as a potential therapeutic strategy. Evidence from the literature outlining these antioxidants and describing their therapeutic effect and contribution to a redox balance has been summarized in Table 1.

## 9. Conclusions and Future Perspectives

In conclusion, while oxidative stress is not among the primary etiologies of β-thalassemia and SCD, it plays a significant role in the pathogenesis of these diseases. The mechanism of oxidative stress development in β-thalassemia and SCD is not only multifactorial in nature but also different. Oxidative stress can be ameliorated with antioxidative treatment modalities that act at various levels. The identification of cytoprotective and antioxidant enzymes and molecules has paved the way for a new era of new pharmacological targets for treating β-thalassemia and SCD. For optimal outcomes, future studies should aim at identifying specific sources of ROS, applying direct and targeted therapy, and improving overall outcomes for patients. A better understanding of the main oxidants and antioxidants involved, and the associated cascade of biological events will provide a better insight to achieve an optimal redox balance.

## Figures and Tables

**Table 1:** Antioxidant therapies in β-thalassemia and SCD patients.

Antioxidant	Subjects	Outcomes
**Vitamins C and E**	• TDT	• Vitamin C plus Vitamin E supplementation promoted antioxidant status [147]
	• NTDT	• Vitamin E supplementation was also associated with a decrease in MDA levels, and amelioration of RBCs osmotic fragility [148]
	• NTDT	• Vitamin E decreased lipid peroxidation in thalassemic RBCs, increased their survival and suppresses hemolysis [149]
	• TDT	• Vitamin E also safely improved total oxidative stress status [150]
**Flavonoids**	• TDT	• Silymarin decreased serum oxidative stress and enhanced serum antioxidant capability [151]
**Curcuminoids**	• NTDT	• Curcuminoids supplementation ameliorated oxidative stress and iron overload [152]
	• TDT	• The combination of curcumoid and green tea extract decreased redox-active iron [153]
**Zinc Supplementation**	• SCD	• Zinc supplementation decreased not only the incidence of infection, but also oxidative stress, inflammatory cytokine generation [154]
**NAC**	• TDT	• NAC was shown to effectively reduce systemic and serum oxidative stress [155,156]
	• SCD	• NAC treatment decreased oxidative stress through a reduced expression of PS expression on the cell membrane, in addition to ↓ levels of advanced glycoxidation end-products [157]
**Alpha-lipoic acid and acetyl-L-Carnitine**	• TDT	• Alpha-lipoic acid supplementation may have an effect on lipid profile and oxidative stress status [158]
	• SCD	• Combination of α-lipoic acid and acetyl-L-carnitine increased glutathione levels and decreased lipid peroxidation and improves plasma redox status [159,160]
**Arginine** **Therapy**	• SCD	• IV arginine therapy increased mitochondrial activity and decreased oxidative stress in children with vaso-occlusive pain [161] • The use of low-dose oral supplementation of L-arginine improved liver function, oxidative stress, plasma arginine concentration and nitric oxide metabolites levels [162]
**Fermented** **papaya preparation**	• TDT and NTDT	• Administration of FPP led to a decrease in ROS generation, membrane lipid peroxidation, and externalization of PS residues concomitant with an increase in GSH levles [163]
**Omega-3 fatty acids**	• SCD	• Administration of omega-3 long-chain polyunsaturated fatty acids can provide an antioxidant protection [164]
**Gum Arabic**	• SCA	• Gum Arabic increased total antioxidant capacity and decreased MDA and H_2_O_2_ levels [165,166]

Abbreviations: TDT: Transfusion Dependent Thalassemia; NTDT: Non-Transfusion Dependent Thalassemia; SCD: Sickle Cell Disease; SCA: Sickle Cell Anemia; MDA: Malondialdehyde; RBC: Red blood cells; NAC: N-acetylcysteine; ROS: Reactive oxygen species; FPP: Fermented papaya preparation; GSH: Glutathione.

## Data Availability

Not applicable.
